# Outbreak of Avian Malaria Associated to Multiple Species of *Plasmodium* in Magellanic Penguins Undergoing Rehabilitation in Southern Brazil

**DOI:** 10.1371/journal.pone.0094994

**Published:** 2014-04-15

**Authors:** Ralph Eric Thijl Vanstreels, Cristiane K. M. Kolesnikovas, Sandro Sandri, Patrícia Silveira, Nayara O. Belo, Francisco C. Ferreira Junior, Sabrina Epiphanio, Mário Steindel, Érika M. Braga, José Luiz Catão-Dias

**Affiliations:** 1 Laboratório de Patologia Comparada de Animais Selvagens, Departamento de Patologia, Faculdade de Medicina Veterinária e Zootecnia, Universidade de São Paulo, São Paulo, São Paulo, Brazil; 2 Associação R3 Animal, Florianópolis, Santa Catarina, Brazil; 3 Departamento de Parasitologia, Instituto de Ciências Biológicas, Universidade Federal de Minas Gerais, Belo Horizonte, Minas Gerais, Brazil; 4 Departamento de Análises Clínicas e Toxicológicas, Faculdade de Ciências Farmacêuticas, Universidade de São Paulo, São Paulo, São Paulo, Brazil; 5 Laboratório de Protozoologia, Departamento de Microbiologia e Parasitologia, Centro de Ciências Biológicas, Universidade Federal de Santa Catarina, Florianópolis, Santa Catarina, Brazil; Bernhard Nocht Institute for Tropical Medicine, Germany

## Abstract

Avian malaria is a mosquito-borne disease caused by *Plasmodium* spp. Avian plasmodia are recognized conservation-threatening pathogens due to their potential to cause severe epizootics when introduced to bird populations with which they did not co-evolve. Penguins are considered particularly susceptible, as outbreaks in captive populations will often lead to high morbidity and rapid mortality. We used a multidisciplinary approach to investigate an outbreak of avian malaria in 28 Magellanic penguins (*Spheniscus magellanicus*) at a rehabilitation center during summer 2009 in Florianópolis, Brazil. Hemosporidian infections were identified by microscopic and molecular characterization in 64% (18/28) of the penguins, including *Plasmodium* (*Haemamoeba*) *tejerai*, *Plasmodium* (*Huffia*) *elongatum*, a *Plasmodium* (*Haemamoeba*) sp. lineage closely related to *Plasmodium cathemerium*, and a *Haemoproteus* (*Parahaemoproteus*) sp. lineage closely related to *Haemoproteus syrnii*. *P. tejerai* played a predominant role in the studied outbreak and was identified in 72% (13/18) of the hemosporidian-infected penguins, and in 89% (8/9) of the penguins that died, suggesting that this is a highly pathogenic parasite for penguins; a detailed description of tissue meronts and lesions is provided. Mixed infections were identified in three penguins, and involved *P. elongatum* and either *P. tejerai* or *P.* (*Haemamoeba*) sp. that were compatible with *P. tejerai* but could not be confirmed. In total, 32% (9/28) penguins died over the course of 16 days despite oral treatment with chloroquine followed by sulfadiazine-trimethoprim. Hemosporidian infections were considered likely to have occurred during rehabilitation, probably from mosquitoes infected while feeding on local native birds, whereas penguin-mosquito-penguin transmission may have played a role in later stages of the outbreak. Considering the seasonality of the infection, rehabilitation centers would benefit from narrowing their efforts to prevent avian malaria outbreaks to the penguins that are maintained throughout summer.

## Introduction

*Plasmodium* spp. are recognized as pathogens that may threaten the conservation of avian communities, particularly when introduced to populations that did not co-evolve with the parasite [1], [2]. In particular, two avian taxa have been shown to be highly susceptible to these parasites, Hawaiian honeycreepers (Drepaniidae) and penguins (Spheniscidae) [3]–[5]. Concerns that this pathogen may be a significant conservation threat for penguins have been raised due to the observation that avian malaria in captive penguins produces rapid and severe outbreaks with as much as 50–80% mortality within few weeks [3], . Four species of *Plasmodium* sp. have been demonstrated to infect penguins: *P.* (*Haemamoeba*) *relictum* [9], *P.* (*Huffia*) *elongatum* [10], *P.* (*Bennettinia*) *juxtanucleare* [11] and *P.* (*Haemamoeba*) *tejerai* [12]. Additionally, there is an anecdotal report of *P.* (*Haemamoeba*) *cathemerium* in penguins [13].

Magellanic penguins (*Spheniscus magellanicus*) are native to Argentina, Chile and the Falkland Islands, and often winter along the coast of Uruguay and Brazil [14], [15]. The susceptibility of this species to malarial parasites has been documented in zoos and rehabilitation centers [3], [8], [12], [16], but not in the wild [17]–[20]. Other studies have failed to detect *Plasmodium* sp. in wild penguins elsewhere in South America or at the Falkland islands, whether in Southern rockhopper (*Eudyptes chrysocome*) [17], [19]–[21], Humboldt (*S. humboldti*) [24]–[27] or Gentoo penguins (*Pygoscelis papua*) [17], [19], [20]. In Galapagos penguins (*Spheniscus mendiculus*), on the other hand, DNA from *Plasmodium* spp. has been detected at the Galapagos Archipelago, even though the parasite was not observed in blood smears [22], [23].

We had previously reported two Magellanic penguins that died due to infection by *P. tejerai* during a mortality outbreak at a rehabilitation center in Florianópolis, Brazil, in early 2009 [12]. In the present paper we follow up on that investigation and provide a broader study on the epidemiology and pathology of *P. tejerai* as well as other hemosporidians involved in the same outbreak. We also discuss the implications of these findings for the rehabilitation and conservation of penguins and other highly susceptible taxa.

## Methods

### Ethics Statement

This study was conducted under approval by the Animal Use Ethics Committee of the University of São Paulo (CEUA-USP 1757/2009) and was authorized by the Brazilian wildlife authority (SISBIO 20825-3).

### Study Population, Sample Collection and Treatment

We examined the Magellanic penguins maintained through austral summer 2008–2009 at the Centro de Triagem de Animais Silvestres do Núcleo de Fauna do IBAMA, Florianópolis, Brazil (27°31′35″S 48°25′44″W). The rehabilitation center is located near a large freshwater lake (Lagoa da Conceição) and is surrounded by Atlantic forest (lowland tropical moist forest), with average annual precipitation of 1524 mm and average annual temperature ranging from 17.0 to 24.8°C [28].

The year 2008 was atypical in presenting unusually high numbers of Magellanic penguins becoming beach-cast along the Brazilian coast [15], and the rehabilitation center received 387 animals. Most penguins were successfully rehabilitated (following protocols by [29]) and released in 2008, however 23 were maintained through summer, and another 5 were received in early 2009 (band numbers 508, 586, 587, 592 and 593); these 28 penguins were the subjects of this study (Table 1). In a previous study [12] we examined in detail two of these individuals (band numbers 506 and 520 are respectively penguins A and B in that study). Metatarsal blood samples were collected on 23/03/2009 (584, 593) and 25/03/2009 (remaining penguins) (Sampling A); penguins that were still alive were sampled again on 05/04/2009 (Sampling B).

**Table 1:** Individual history and diagnostic results for the studied Magellanic penguins (*Spheniscus magellanicus*).

Bandno.	PCR(A)	PCR(B)	Phylogeneticgroup/lineage	Blood smear(A)	Blood smear(B)	Histo-pathology	Hemosporidianspecies
503*	–	Positive	A	*Plasmodium* sp.	*P.* (*Haemamoeba*) sp.	–	[*P. tejerai*]
504	Positive	Negative	Seq. unsuccessful	Negative	Negative	–	Undetermined
506*	Positive	–	A	*P. tejerai*	–	Positive	*P. tejerai*
507	Positive	Negative	A	Negative	Negative	–	[*P. tejerai*]
508*	Positive	Positive	A+C	*P. elongatum+P.* (*Haemamoeba*) sp.	*P. elongatum+P. tejerai*	–	*P. elongatum+P. tejerai*
509	–	Negative	–	Negative	Negative	–	–
510	–	Negative	–	–	Negative	–	–
511	Positive	Positive	A	–	Negative	–	[*P. tejerai*]
512	Positive	Negative	A	Negative	Negative	–	[*P. tejerai*]
513	–	Negative	–	Negative	Negative	–	–
514	–	Negative	–	Negative	Negative	–	–
515	–	Negative	–	Negative	Negative	–	–
516	–	Positive	B	Negative	*Plasmodium* sp.	–	[*P.* (*Haemamoeba*) sp.]
517	–	Negative	–	*P. elongatum+P.* (*Haemamoeba*) sp.	Negative	–	*P. elongatum+P.* (*Haemamoeba*) sp.
518*	Positive	–	A	–	Negative	–	[*P. tejerai*]
520*	Positive	–	A	*P. tejerai*	–	–	[*P. tejerai*]
584*	–	–	–	*P. tejerai*	–	Positive	[*P. tejerai*]
585	–	Negative	–	Negative	Negative	–	–
586	–	Positive	D	Negative	Negative	–	[*H.* (*Parahaemoproteus*) sp.]
587*	–	–	–	Negative	–	–	–
588	Positive	Negative	Seq. unsuccessful	*P. elongatum+P.* (*Haemamoeba*) sp.	Negative	–	*P. elongatum+P.* (*Haemamoeba*) sp.
589*	Positive	Negative	A	Negative	Negative	–	[*P. tejerai* ]
590	Positive	–	A	–	*P.tejerai*	–	*P. tejerai*
592	–	Negative	–	Negative	Negative	–	–
593*	–	–	–	*P. tejerai*	–	Positive	*P. tejerai*
16437	Positive	Negative	A	Negative	Negative	–	[*P. tejerai*]
16445	–	Negative	–	Negative	–	–	–
16682	–	–	–	–	Negative	–	–

Taxonomic names within brackets indicate the taxon to which the species is presumed to correspond on the basis of phylogenetic analyses. Asterisks indicate individuals that died during the outbreak.

Veterinarians monitored penguins daily throughout the rehabilitation process, and any clinical signs or abnormalities were recorded. After avian malaria was diagnosed, penguins were orally treated with chloroquine on 01/04/2009 (10 mg/kg at hour zero; 5 mg/kg at hours 6, 18 and 24) until 10/04/2009 (5 mg/kg q24 h), then with sulfadiazine-trimethoprim (40 mg/kg q24 h) from 11/04/2009 to 20/04/2009.

### Laboratory Procedures

Two thin blood smears were performed immediately after each blood collection, dried at room temperature, and fixed with methanol. Two heparin capillaries were also collected immediately after collection. The remaining blood was stored in EDTA flasks and refrigerated (4°C) for 2–4 hours, centrifuged at 1500 G for 10 minutes, supernatant fluid was separated and both blood cells and plasma were frozen (−20°C). Packed Cell Volume (PCV) was determined through centrifugation in heparin capillaries at 16000 G for 5 min.

Blood smears were Giemsa-stained within a week of collection; at least 300 microscopic fields were examined for parasites in each smear, under 1000× magnification (>50.000 erythrocytes). Blood parasites were morphologically characterized [30] and quantified [31], [32]; differential parasite counts (trophozoites, meronts, macrogametocytes and microgametocytes) were conducted for 100 parasites or for all parasites observed. Differential leukocyte counts (heterophils, eosinophils, basophils, lymphocytes, monophils) were conducted for 200 leukocytes per slide and Early Erythrocytic Lineage Cells (EELC) were estimated as a percentage of all red blood cells. Heterophil-to-Lymphocyte Ratio (HLR) was calculated. Blood smear examination was blind to PCR results and vice-versa.

Deceased animals were examined within 12 to 24 h post-mortem, and organs and tissues were fixed in 10% buffered formalin and embedded in paraffin. Sections of 5 µm were obtained, stained with hematoxylin-eosin and examined under light microscopy.

### Cytochrome b Amplification and Phylogenetic Analysis

Frozen red blood cells in EDTA and frozen tissue samples were used for molecular analyses. DNA extraction was conducted using the DNEasy Blood and Tissue Kit (#69506, Qiagen, Venlo, Netherlands) following the manufacturer’s recommended protocol. DNA extraction was verified and quantified through UV spectrophotometry (Nanodrop 2000, Thermo Fisher Scientific, Wilmington, DE, USA). A nested PCR targeting a 480 bp fragment of the cytochrome b (*cyt-b*) mitochondrial gene of *Haemoproteus* sp. and *Plasmodium* sp. was used [33] (each reaction had 25 µL, with 75 ng of sample DNA; first reaction with primers HaemNFI and HaemNR3, second reaction with primers HaemF and HaemR2). GoTaq Green Master Mix (M7122, Promega, Madison, USA) was used for both reactions. Blood samples from chicken experimentally infected with *Plasmodium gallinaceum* or raised in arthropod-free environments were used as positive and negative controls, respectively. Amplification products were visualized in 6% silver nitrate-stained polyacrylamide gels and 2% agarose gel [34], [35].

PCR amplification products of positive samples were purified with Polyethylene glycol 8000. Bi-directional sequencing with dye-terminator fluorescent labeling was performed through automated sequencing (ABI Prism 3100, Applied Biosystems, Foster City, USA). Forward and reversed chromatograms were aligned and sequences were revised and edited using DNABaser (Heracle BioSoft SRL, Pitesti, Romania); *cyt-b* gene resulting sequences were deposited in GenBank (Table S1).

Phylogenetic relationships among the hemosporidian lineages identified in this study and related hemosporidian parasites were inferred by using *cyt-b* gene sequences from MalAvi database [36] for which morphospecies had been determined using blood smears (Table S1). Because GenBank contains misidentified sequences of avian hemosporidians [37], only lineages for which extensive blood smear morphology data was available were included when BLAST search indicated high identity and query cover (≥95%) with the sequences obtained in this study [38]–[42]. In addition, we included hemosporidian lineages recovered from penguins at the Galapagos Archipelago [22], [23], São Paulo Zoological Park [8] and Australia [43]. Sequences were aligned (File S1) using ClustalW [44] as implemented in MEGA 5.2.2 [45]. A maximum likelihood phylogenetic tree for the parasite sequences was produced using MEGA 5.2.2 with the GTR+gamma model of nucleotide evolution (selected using ModelTest [46]), and 1000 bootstrap replications. There is on-going debate on which taxon should be used to root phylogenetic trees of hemosporidians [47], we used *L. schoutedeni* merely for visualization purposes.

### Statistical Analyses

Two-sample t-tests were used to compare parasitemia and hematological parameters in the blood smears of penguins that were deceased and those that survived. Paired t-tests were used to compare hematological parameters (heterophils, eosinophils, basophils, lymphocytes, monocytes, EELC, HLR, PCV) between positive and negative individuals and between Sampling A and B, and mean difference tests were used to determine whether these sampling date differences followed distinct patterns between positive or negative individuals. Linear regression was used to determine whether correlations existed between parasitemia and hematological parameters. ANOVA was used to compare the percentage of parasite forms (trophozoites, meronts, macrogametocytes, microgametocytes) in the blood smears of penguins infected by different parasite lineages. Fisher’s exact test was used to compare mortality between individuals infected with *P. tejerai* and other lineages. Significance level was 0.05 for all tests, and the Dunn-Šidák correction was applied for multiple comparisons.

## Results

### Clinical Signs and Necropsy Findings

In early March 2009, 28 Magellanic penguins were at the rehabilitation center, and were considered clinically healthy. Penguins died on 23/03/2009 (band numbers 584 and 593), 27/03/2009 (506), 29/03/2009 (518, 520, 589), 03/04/2009 (587), and 07/04/2009 (503, 508). All deceased penguins had good body condition, normal appetite and behavior on the days preceding their deaths, and shared similar necropsy findings: hydropericardium, cardiomegaly, lung congestion, hepatomegaly and splenomegaly.

### Hemosporidian Detection

Table 1 details PCR, blood smear, histopathology and gene sequencing results for the 28 penguins present at the rehabilitation center in April 2009. Eighteen penguins had one or more positive samples (64.3%; 18/28) whether through blood smears alone (50%; 2/4) or using a combination of blood smears and nested PCR (66.7%; 16/24). Of the 14 samples positive to PCR for which blood smears were also available, only 7 were identified as positive in the corresponding blood smears (50%). All PCR-negative samples were negative to parasites in the corresponding blood smears. No blood parasites other than hemosporidians were observed.

All twelve penguins tested by PCR at Sampling A were positive (100%). Nine of these PCR-positive penguins also had blood smears examined: 4 were positive (44.4%). An additional 14 penguins that had not been tested by PCR had blood smears examined at Sampling A, of which 4 were positive (28.6%). Five of the 20 penguins tested by PCR at Sampling B were positive (25%); of these, 3 had their blood smears identified as positive (60%). An additional 3 penguins that had not been tested by PCR had blood smears examined at Sampling B, of which one was positive (33.3%). As a result, 16 of the 26 penguins had positive results at Sampling A (61.5%) and 6 of the 23 penguins had positive results at Sampling B (26.1%).

### Parasite Identification and Phylogeny

**Figure 1:**
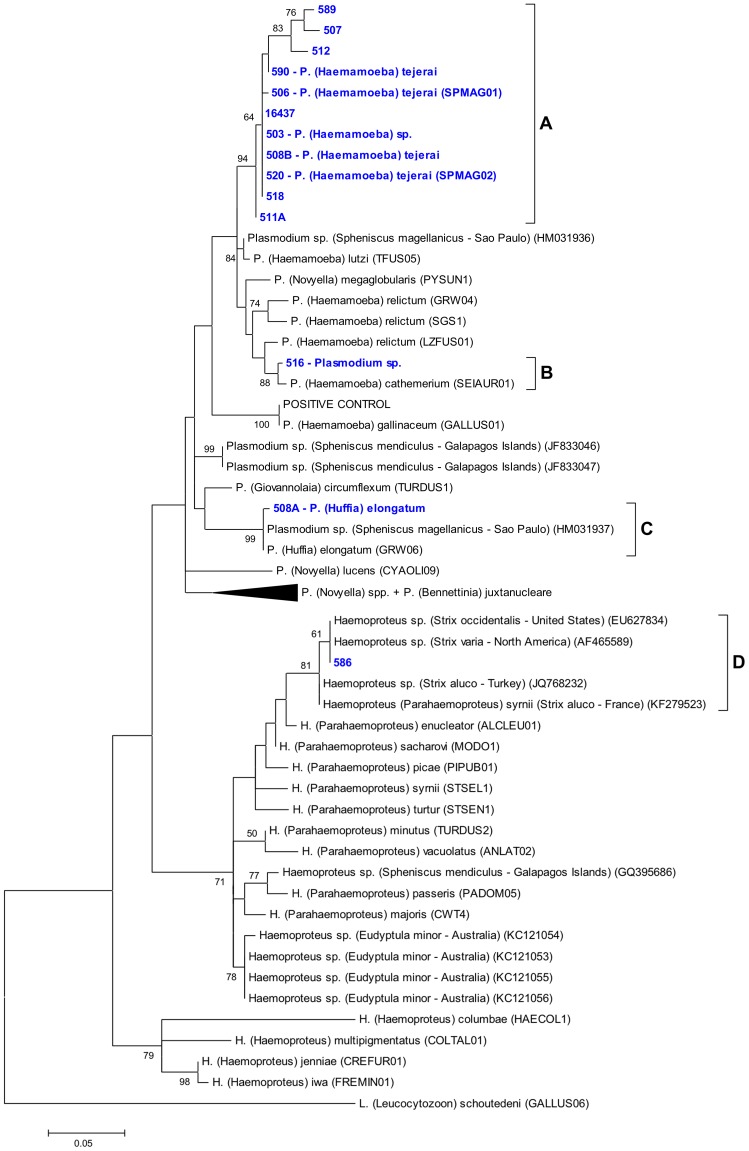
Maximum likelihood phylogenetic tree of the mitochondrial cytochrome b gene of the studied hemosporidian lineages. Lineages identified in this study are emphasized in blue. When available, information on the morphospecies observed on the corresponding blood smear is provided. Branch lengths are drawn proportionally to evolutionary distance (scale bar is shown). Lower bootstrap values (<50) are omitted.

Mitochondrial cytochrome b gene sequences were obtained for 17 samples from 13 penguins. Phylogenetic analysis revealed four phylogenetic groups/lineages, three *Plasmodium* sp. (A, B and C) and one *Haemoproteus* sp. (D) (Figure 1). Two penguins had two samples that yielded two different sequences each (band numbers 508 and 511); one of these (511B) had eight nucleotide polymorphisms (mixed peaks on the sequencing chromatogram) and was excluded from phylogenetic analyses. Two samples were PCR-positive but failed to produce adequate sequencing results (504 and 588). Table 2 summarizes evolutionary distance within these groups and published lineages.

**Table 2:** Estimates of evolutionary distance (% expected base substitutions per site) of cytochrome b mitochondrial gene sequences of hemosporidians identified in penguins in this study (1–6) and the literature (7–10), and reference lineages from the MalAvi database (11–19).

		2	3	4	5	6	7	8	9	10	11	12	13	14	15	16	17	18	19
**1**	507 (Group A)	3.2	6.9	9.1	12.3	15.4	8.1	11.9	9.5	9.5	8.1	9.5	10.0	11.9	9.9	14.1	15.7	16.3	27.7
**2**	520 (Group A) (SPMAG02)		3.5	5.6	8.6	11.4	4.7	8.2	5.9	5.9	4.7	6.0	6.4	8.2	6.4	10.3	11.7	12.3	22.7
**3**	511A (Group A)			5.2	7.7	10.4	2.7	7.3	3.9	3.9	3.1	6.0	5.6	7.3	6.0	9.0	10.0	10.9	21.1
**4**	516 (Group B)				6.5	9.6	4.4	6.0	6.0	6.0	4.3	4.4	1.5	6.0	5.6	9.0	10.9	11.4	19.7
**5**	508A (Group C)					9.6	6.4	0.4	6.0	6.0	6.5	6.5	6.9	0.4	6.5	9.9	12.8	12.1	18.1
**6**	586 (Group D)						11.0	9.2	8.3	8.3	11.9	11.5	10.1	9.2	9.6	14.3	3.9	10.8	20.7
**7**	*Plasmodium* sp.(*S. magellanicus* - HM031936)							6.0	4.0	4.0	0.8	5.2	4.3	6.0	6.0	8.6	10.9	11.8	18.6
**8**	*Plasmodium* sp.(*S. magellanicus* - HM031937)								5.6	5.6	6.0	6.0	6.5	0.0	6.0	9.4	12.3	11.7	18.6
**9**	*Plasmodium* sp.(*S. mendiculus* - JF833046)									0.0	3.9	6.0	6.5	5.6	4.8	7.2	10.5	10.8	20.7
**10**	*Plasmodium* sp.(*S. mendiculus* - JF833047)										3.9	6.0	6.5	5.6	4.8	7.2	10.5	10.8	20.7
**11**	*P. (Haemamoeba)* *lutzi* (TFUS05)											5.2	4.3	6.0	6.0	7.7	11.8	12.2	18.7
**12**	*P. (Haemamoeba)* *relictum* (GRW04)												4.8	6.0	6.0	9.5	12.9	12.8	20.7
**13**	*P. (Haemamoeba)* *cathemerium* (SEIAUR01)													6.5	4.8	8.6	10.5	11.4	19.8
**14**	*P. (Huffia) elongatum*(GRW06)														6.0	9.4	12.3	11.7	18.6
**15**	*P. (Giovannolaia)* *circumflexum* (TURDUS1)															10.8	10.9	10.9	21.6
**16**	*P. (Bennettinia)* *juxtanucleare* (GALLUS03)																15.8	13.6	23.2
**17**	*H. (Parahaemoproteus)* *syrnii* (STSEL1)																	12.6	22.1
**18**	*H. (Haemoproteus)* *iwa* (FREMIN01)																		22.2
**19**	*L. (Leucocytozoon)* *schoutedeni* (GALLUS06)																		

Values lower than 4.0 are underlined.

**Figure 2:**
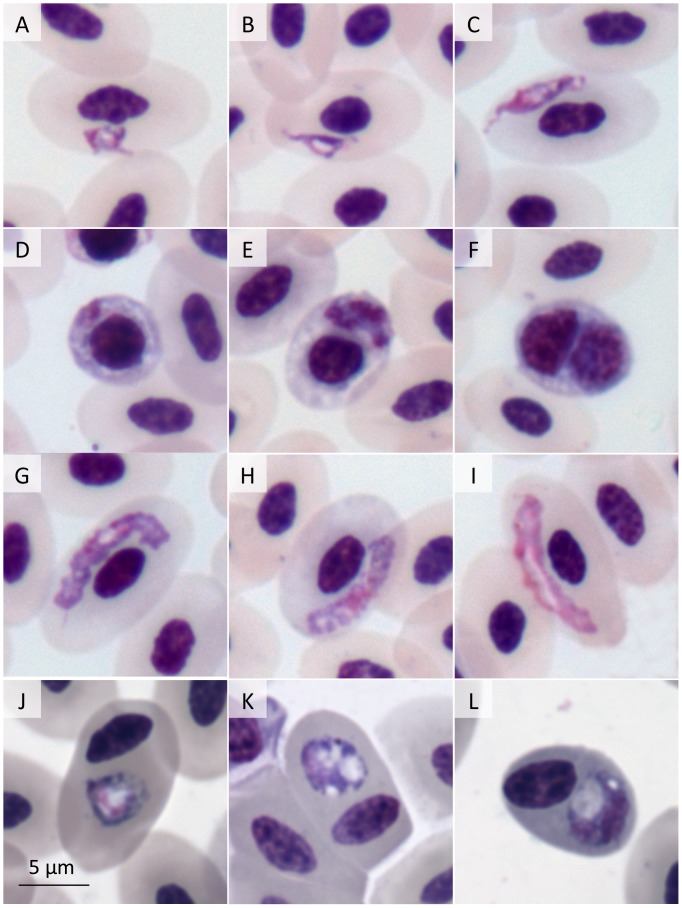
Blood parasites in Giemsa-stained blood smears from Magellanic penguins (Spheniscus magellanicus). *Plasmodium* (*Huffia*) *elongatum* (508B) trophozoites (a–d), meronts (e,f), macrogametocytes (g,h) and microgametocyte (i); chloroquine-degenerated *Plasmodium* (*Haemamoeba*) sp. (503B) (j–l).

Phylogenetic group A comprised sequences obtained from 13 samples (clade bootstrap value = 94%; average evolutionary divergence within group = 0.007 base substitutions per site), and had some of its samples morphologically identified as *P. tejerai* (for detailed descriptions and photomicrographs see [*12*]). Lineage B was recovered only from one penguin (516), and its morphospecies could not be determined as only early trophozoites were present in the blood smear; this lineage formed a clade with a known *P. cathemerium* lineage (bootstrap value = 88%; evolutionary divergence = 0.015 base substitutions per site; 98.7% sequence identity). Lineage C was recovered only from one penguin (508) and was morphologically confirmed as *P. elongatum* (Figure 2a–i); this lineage formed a strongly supported clade (bootstrap value = 99%; average evolutionary divergence within group = 0.004 base substitutions per site) with a known *P. elongatum* lineage (98.5% sequence identity) and with a *Plasmodium* sp. lineage previously identified in Magellanic penguins (98.0% sequence identity). Lineage D was recovered only from one penguin (586) and its morphospecies could not be determined because no parasite forms were present in the blood smear; this lineage formed a clade (bootstrap value = 81%; average evolutionary divergence within group = 0.009 base substitutions per site) with *H.* (*Parahaemoproteus*) sp. lineages obtained from earless owls (*Strix* spp.) in the Northern Hemisphere, including a known *Haemoproteus syrnii* lineage (96.8% sequence identity). The positive control used for the nested PCR reactions was identical to a known *P. gallinaceum* lineage (100% sequence identity) and unrelated to the study lineages.

Mixed infections were identified in three individuals (band numbers 508, 517 and 588), and involved *P. elongatum* and either *P. tejerai* or *P.* (*Haemamoeba*) sp. that were compatible with *P. tejerai* but could not be undoubtedly confirmed. In two cases (503 and 516) the parasites observed at Sampling B were severely degenerated, presumably as a result of chloroquine treatment, producing atypical forms that at times bore little resemblance to typical hemosporidian forms (Figure 2j–l).

Nine of 28 penguins (32.1%) died during the study period. Of these, eight (88.9%) had been positive to hemosporidians, i.e. 8 of 18 penguins that obtained positive results died during the study period (44.4%). All hemosporidian-positive deceased penguins were infected with *P. tejerai*, one of which was co-infected with *P. elongatum*. Even though 58.3% of the penguins confirmed to be infected only with *P. tejerai* died (7/12), this was not significantly different to the mortality observed among individuals infected or co-infected with other lineages (16.7%; 1/6) (P = 0.152).

Parasitemia ranged from 0.001% to 62% (Mean ± S.D. = 8.66% ±18.83%). Even though parasitemia was apparently higher in penguins that died (12.97% ±22.22%) than in those that survived (0.06% ±0.10%), this difference did not reach significance (t = 1.642, df = 7, P = 0.072). Trophozoites (P<0.001, R^2^ = 0.787), macrogametocytes (P<0.001, R^2^ = 0.854) and microgametocytes (P = 0.008, R^2^ = 0.585) were unevenly distributed among parasite lineages (Table 3, Table S2), but not meronts (P = 0.390).

**Table 3:** Hematological results and quantification of blood parasites in blood smears (Mean ± S.D.).

	*P. tejerai*(lineage A)	*Plasmodium* sp.(lineage B)	*P. elongatum*+*Plasmodium* sp.	*Haemoproteus* sp.(lineage D)	Negative to hemosporidians
**Parasite forms**					
Trophozoites	80.3% ±13.6%	100%	21.7% ±17.3%	–	–
Meronts	13.3% ±11.6%	0%	5.5% ±9.7%	–	–
Macrogametocytes	4.6% ±4.2%	0%	33.7% ±8.5%	–	–
Microgametocytes	2.1% ±1.9%	0%	39.0% ±25.0%	–	–
Parasitemia	14.5% ±23.5%	<0.001%	5.6% ±6.1%	–	–
Sample size (n)	7	1	3	–	–
**Hematology**					
Heterophils	48.1% ±17.2%	70.5% ±12.0%	45.3% ±16.9%	53.5% ±24.7%	51.1% ±14.7%
Eosinophils	1.8% ±1.5%	0%	2.0% ±1.1%	2.0% ±1.4%	2.7% ±1.8%
Basophils	0.3% ±0.5%	0.5% ±0.7%	0.2% ±0.4%	0%	0.3% ±0.7%
Lymphocytes	49.0% ±15.7%	29.5% ±12.0%	51.8% ±14.9%	44.0% ±24.0%	46.0% ±13.9%
Monocytes	1.1% ±1.8%	0%	1.2% ±2.0%	1.5% ±0.7%	0.3% ±0.6%
HLR	1.24±0.74	2.66±1.43	1.10±0.99	1.61±1.43	1.35±1.19
EELC	13.6% ±9.5%	25% ±7.0%	14.3% ±12.8%	7.5% ±3.5%	9.2% ±4.8%
PCV	38.2% ±3.6%	22.0% ±2.8%	30.8% ±7.8%	39.5% ±0.7%	36.9% ±6.1%
Sample size (n)	17	2	6	2	16

### Hematology and Pathology

Table 3 summarizes the average hematological results for blood smears of penguins infected by different hemosporidian lineages (raw data are provided in Table S2). No significant differences were observed in these hematological parameters between Sampling A and B, whether they were positive to hemosporidians or not (all P>0.05), nor between penguins that died or those that survived (all P>0.05). Parasitemia was positively correlated to lymphocytes (P = 0.022, R^2^ = 0.364), monocytes (P = 0.043, R^2^ = 0.285) and PCV (P = 0.034, R^2^ = 0.642) and was negatively correlated to heterophils (P = 0.026, R^2^ = 0.348); no significant correlation was observed between parasitemia and basophils, eosinophils, EELC or HLR (all P>0.05).

**Figure 3:**
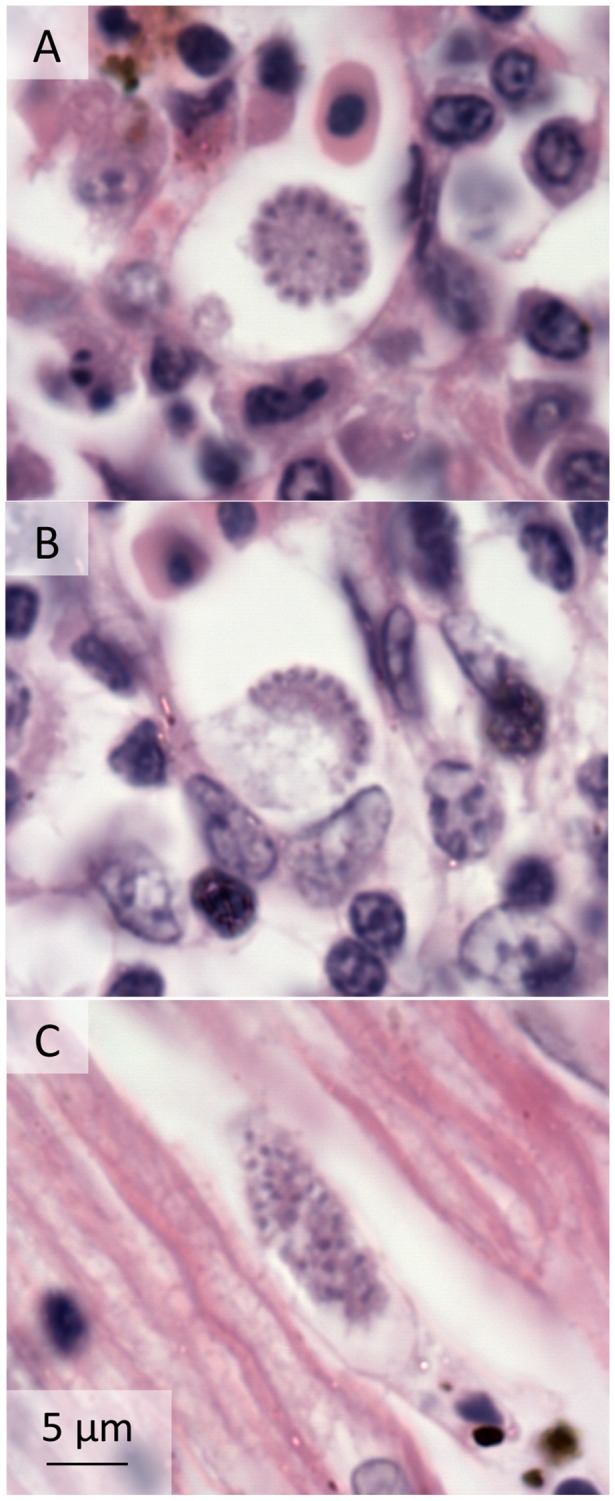
Tissue meronts of *Plasmodium* (*Haemamoeba*) *tejerai* in tissues of Magellanic penguins (*Spheniscus magellanicus*). Hematoxilin-Eosin, penguin 584: liver (a), parathyroid (b) and heart (c).

Histopathology was examined for three individuals (506, 584, 593), all of which were infected only with *P. tejerai*. Tissue meronts were occasionally present in macrophages, but were overall most common in endothelial cells. Meronts were most frequent in the heart and kidneys, moderately frequent in the lungs and colon, and were rare in other tissues (spleen, liver, testicles, brain, duodenum, pancreas, gastrointestinal tract-associated lymphoid tissue, thyroid, parathyroid). Penguin 584 was considered to have a higher number of tissue meronts (approx. 2–3 per 10 high magnification microscope fields) than penguins 506 and 593 (less than 1 per 10 fields), whereas ante-mortem parasitemia was considerably higher in 506 (62%) and 593 (30%) than in 584 (7.4%). Tissue meronts ranged between 10 and 80 µm in largest diameter, most frequently from 15 to 30 µm. They were often round or oval, with merozoites distributed near the external surface forming structures resembling arcs (Figure 3a) or semi-arcs (Figure 3b), or were elongated and contained randomly scattered merozoites (Figure 3c). The envelope of tissue meronts was mildly eosinophilic, thin and poorly defined; merozoites were round and densely stained with size of approximately 1 µm in diameter. Each meront contained tens to hundreds of visible merozoites, although generally less than 20 merozoites were visible in 3 µm-thick histological sections.

Major pathological processes included: moderate to severe diffuse interstitial granulocytic pneumonia, moderate to severe pulmonary edema and congestion, severe acute necrotizing splenitis, moderate multifocal to coalescent mixed or predominantly mononuclear necrotizing hepatitis, mild to moderate multifocal interstitial subacute nephritis, moderate diffuse iron deposits in the lungs, liver and spleen, mild to moderate spleen histiocytosis, mild to moderate multifocal necrosis of gut-associated lymphoid tissues, mild diffuse granulocytic myocarditis, multifocal to coalescent areas of cardiomyolisis. Penguin 584 also had diffuse acute vasculitis associated to the formation of intravascular cloths. Death probably resulted from cardiorespiratory insufficiency secondary to pneumonia and, in the case of penguin 584, in association with disseminated vasculitis and intravascular coagulation. No signs or lesions suggestive of viral, bacterial or fungal co-infections were observed.

## Discussion

Penguins are notoriously susceptible to avian malaria, and Magellanic penguins are not an exception [25]. Fix and colleagues [3] reported three successive malaria outbreaks leading to the mortality of 15.2%, 51.3% and 57.9% of Magellanic penguins captive at a zoo in Iowa, USA (cumulative mortality = 82.6%; n = 46). Bueno and colleagues [8] reported an outbreak with 80% infection rate and 60% mortality of Magellanic penguins captive at a zoo in São Paulo, Brazil (n = 5). In this study, we observed 60.7% infection rate and 32.1% mortality of Magellanic penguins undergoing rehabilitation during summer in Florianópolis, Brazil (n = 28).

The use of chloroquine and primaquine for the treatment of avian malaria in penguins was first proposed by Stoskopf and Beier [48], and is based on the combined antiprotozoal effect of chloroquine on the circulating stages and of primaquine on the tissue stages [49]. Because we were unable to administer primaquine the poor performance of the drug treatment in this outbreak was not entirely unexpected. However, Fix et al. and Bueno et al. [3], [8] did administer the combined drug treatment and still were confronted with high mortality rates, suggesting a poor efficacy of these drugs, as has also been reported for other penguin species [50], [51]. Our observation of degenerated erythrocytic parasites in blood smears of some of the penguins subjected to five days of chloroquine treatment suggests an effective antiprotozoal action of this drug on circulating parasites. Unfortunately, because histopathology was conducted only for individuals that died before drug treatment was initiated, we could not evaluate its effects on the parasites’ tissue stages, if any.

Four lineages of hemosporidians were involved in the outbreak: *Plasmodium* (*Haemamoeba*) *tejerai*, an unidentified *Plasmodium* (*Haemamoeba*) sp. (“lineage B”), *Plasmodium* (*Huffia*) *elongatum*, and an unidentified *Haemoproteus* (*Parahaemoproteus*) sp. Furthermore, because four lineages could not be conclusively identified, it is possible that additional hemosporidian lineages/species were present.

The finding of *P. tejerai* in Magellanic penguins corresponds to the first cases of this parasite other than its original description in domestic turkeys (*Melleagris gallopavo*) in Venezuela [52]. *P. tejerai* played a predominant role in the studied outbreak, being identified in 72.2% of the hemosporidian-infected penguins. Furthermore, the observation that 61.6% of the penguins diagnosed with *P. tejerai* have died suggests this is a highly pathogenic parasite for penguins.

Necropsy and histopathology indicated the death of *P. tejerai*-infected penguins was associated with a significant impairment of cardiorespiratory function and, at least in one case, disseminated vasculitis and intravascular clotting. These findings are not unlike those observed in other malaria outbreaks in penguins [3], [10], [53]–[56], and are compatible with the mechanisms of avian malaria pathogenesis described in other birds [30], [57], [58]. The semi-arc or arc-shaped tissue meronts observed in these infections seem unusual and distinct from those classically reported in avian hemosporidians [30], [59], [60], and could be a characteristic morphological feature of *P. tejerai*, meriting further investigation. It should be noted that such tissue meront morphology is paralleled by the parasite’s morphology in erythrocytes: rosette-shaped erythrocytic meronts in which the merozoites are distributed in an arc near the external border of the parasite’s cytoplasm are one of the key morphological characteristics for the identification of *P. tejerai* in blood smears [12].

*Plasmodium* sp. lineage B was identified in one penguin and could not be identified on the basis of parasite morphology on blood smears because only early trophozoites were present, however sequencing data suggests it belongs to the subgenus *Haemamoeba* and is closely related to *P. cathemerium*. In a review on the parasitology of zoo animals, Luera-Carbo [13] mentions that *P. cathemerium* infects king penguins (*Aptenodytes patagonicus*), presumably at the Barcelona Zoo, Spain; however, because no details were provided on the methods and criteria for species identification, this record cannot be confirmed. It should be considered that the identification of *P. cathemerium* in penguins merits particular caution since there is significant morphological similarity between this parasite and *P. relictum* [30], a species frequently reported in penguins worldwide [3], [7], [9], [48], [61]. Furthermore, the morphology of avian hemosporidians is known to vary considerably when transmitted to different hosts, contributing to confusion in their identification [30], [62]. Because we were not able to confirm the species identity through morphological analysis, we consider that additional evidence is necessary before it may be conclusively demonstrated that *P. cathemerium* infects penguins.

*P. elongatum* is a cosmopolitan parasite that may infect a broad variety of avian hosts, particularly Passeriformes [30], and is not uncommon in penguins captive in North America [48], [50], [54], [63]. Similarly to the reports from North American zoos, *P. elongatum* played a secondary role and did not produce high mortality during malaria outbreaks in penguins. All three cases of *P. elongatum* infection herein examined had a second *Plasmodium* (*Haemamoeba*) sp. lineage, whether confirmed as *P. tejerai* or not; this is also not unusual as co-infection by *P. elongatum* and *P.* (*Haemamoeba*) sp. has been frequently reported in captive penguins in North America [10], [48], [51], [63].

Bueno and colleagues [8] identified two lineages of *Plasmodium* sp. in Magellanic penguins at São Paulo Zoo, and used BLAST to determine high sequence identity with two lineages that had been registered on GenBank as *P. relictum*, AY733088 and AY733089. However, Valkiūnas and colleagues [37] indicated that AY733088 had been misidentified and probably corresponds to *P. elongatum* instead. Our results support this interpretation, and suggest that the two lineages identified by Bueno and colleagues [8] correspond to two distinct species, of which one (HM031937) corresponds to *P. elongatum* and the other (HM031936) corresponds to a *Plasmodium* (*Haemamoeba*) sp. lineage closely related to *P. lutzi*. This corroborates the warnings that sequence identity in GenBank/BLAST databases search is not an adequate method to conclusively identify avian hemosporidians without complementary morphological analysis [36], [37], [64]. On the other hand, our results indicate there is no direct relationship between the *Plasmodium* sp. lineages herein detected and those found in Galapagos penguins, which remain unidentified.

Sequencing results indicate that a *Haemoproteus* (*Parahaemoproteus*) sp. lineage was present in one penguin. *Haemoproteus* sp. is a common avian hemosporidian that is seldom considered pathogenic [30], [57]. Only twice have these parasites been recorded in penguins: DNA from this parasite has been detected in live Galapagos penguins at Galapagos Archipelago [22] and in deceased little penguins (*Eudyptula minor*) in Western Australia [43]. All *Haemoproteus* spp. infections in penguins were identified through gene sequencing analysis, as no circulating stages or only early trophozoites were detected in blood smears. Similarly, we failed to detect circulating parasites in the blood smears corresponding to the PCR-positive sample sequenced as *Haemoproteus* sp. This may indicate a very low parasitemia, insufficient to allow for blood smear detection [65], or could correspond to the detection of DNA from recently inoculated sporozoites which may or not succeed to produce infection [23], [66]. Phylogenetic analysis indicated the *Haemoproteus* sp. lineage identified in this study was not closely related to those previously identified in penguins, and instead was neatly clustered with those found in earless owls (*Strix* spp.) in Europe and North America, including a *Haemproteus syrnii* lineage. Three *Strix* spp. are native to the Florianópolis region (*S. huhula*, *S. hylophila*, and *S. virgata*) [67], [68] and could have served as reservoirs of infection.

Parasitemia was positively correlated to lymphocyte and monocyte counts and inversely correlated to heterophil counts, corroborating the observation that avian malaria elicits a predominantly mononuclear leukocytosis in penguins [63]. On the other hand, parasitemia was positively correlated to PCV, which contradicts classical findings that *Plasmodium*-induced hemolysis results in decreased PCV [57], [58], [63]; this could be explained by a false elevation of PCV due to dehydration as a result from anorexia and febrile syndrome.

Avian-infecting *Plasmodium* spp. are exclusively transmitted by mosquitoes (Culicidae), and gene sequencing data indicates all *Haemoproteus* spp. identified to date in penguins belong to the subgenus *Parahaemoproteus* [22], [43], which is transmitted by biting midges (Ceratopogonidae) [30]. Both mosquitoes and biting midges are abundant in the studied rehabilitation center during summer (subj. obs.). This, combined to the observation that all deaths in the studied malaria outbreak occurred within a 16-days period during summer whereas most penguins had been in the same facility for several months, corroborates the interpretation that infection occurred during rehabilitation, as opposed to a recrudescence/exacerbation of infections acquired in the wild. An alternative, less likely in our opinion, is that one or more penguins relapsed from an asymptomatic *Plasmodium* infection and transmitted the parasite to the other penguins. Although transmission from mosquitoes infected while feeding on local native birds to penguins is likely responsible for the initiation of these outbreaks, the fact that some penguins had large numbers of gametocytes suggests that penguin-mosquito-penguin transmission could play a role in later stages of the outbreak. Early diagnosis, quarantine and treatment of affected individuals could therefore be beneficial to the mitigation of outbreaks in penguins undergoing rehabilitation. Because these parasites may be transmitted through the inoculation of blood from an infected bird to another [30], penguin rehabilitators should also take care to prevent iatrogenic transmission.

Our findings corroborate the strong seasonality of avian malaria outbreaks in captive penguins, which are consistently concentrated in the late summer and early autumn across the world [3], [6]–[8], [16], [56], [63]. Rehabilitation centers would thus benefit from narrowing their malaria prevention efforts to the minority of Magellanic penguins that are maintained throughout summer, as opposed to the majority of penguins admitted and released during winter and early spring [14], [15].

## Supporting Information

Table S1 GenBank ascension numbers of the sequences analyzed. Taxonomic names within brackets indicate the taxon to which the species is presumed to correspond on the basis of phylogenetic analyses.
(XLS)

Table S2 Raw hematological results.
(XLS)

File S1 Sequence alignment used in the phylogenetic analysis.
(FAS)
