# Correction: Outbreak of Avian Malaria Associated to Multiple Species of *Plasmodium* in Magellanic Penguins Undergoing Rehabilitation in Southern Brazil

**DOI:** 10.1371/journal.pone.0103670

**Published:** 2014-07-21

**Authors:** The PLOS ONE Staff

In the Funding section, the funder National Institute of Science and Technology, Genetic and Health Information of the Brazilian Livestock (INCT-IGSPB) is missing. The correct funding information is as follows: This study was supported by the São Paulo Research Foundation (FAPESP 2009/53956-9, 2010/51801-5), Minas Gerais Research Foundation (FAPEMIG), Brazilian Federal Agency for the Support and Evaluation of Graduate Education (CAPES), National Counsel of Technological and Scientific Development (CNPq) and National Institute of Science and Technology, Genetic and Health Information of the Brazilian Livestock (INCT-IGSPB). The funders had no role in study design, data collection and analysis, decision to publish, or preparation of the manuscript.
