# Correction: Outbreak of Avian Malaria Associated to Multiple Species of *Plasmodium* in Magellanic Penguins Undergoing Rehabilitation in Southern Brazil

**DOI:** 10.1371/journal.pone.0116554

**Published:** 2014-12-22

**Authors:** Ralph Eric Thijl Vanstreels, Cristiane K. M. Kolesnikovas, Sandro Sandri, Patrícia Silveira, Nayara O. Belo, Francisco C. Ferreira, Sabrina Epiphanio, Mário Steindel, Érika M. Braga, José Luiz Catão-Dias

Regarding the findings of Vanstreels et al. (2014), later analyses revealed that the blood sample of a tropical screech owl (Megascops choliba) that had been analyzed 4 weeks earlier at the same laboratory yielded a Haemoproteus sp. cyt-b sequence that was 100% identical to that obtained for penguin 586. This owl had been sampled in 2009 at Carapicuíba, SP, Brazil (23°34’S 46°52’W), and blood smears confirmed the infection by Haemoproteus syrnii (3.83 parasites per 10000 erythrocytes).

We reason that (a) H. syrnii is thought to be host-specific to the order level and had never been reported in non-Strigiformes hosts [2], (b) no parasites were identified in the blood smear of penguin 586 whereas they were abundant in the blood of the owl, and (c) the nested PCR method employed uses a total 55 amplification cycles [3], which may theoretically amplify each fragment up to 255 times and thus has a significant probability of contaminating the laboratory equipment and facilities.

We therefore conclude that the positive result obtained for penguin 586 was a false positive due to contamination from the owl sample, and thus the Haemoproteus lineage identified in our study (“lineage D”; GenBank KJ575554) was not identified in penguins.

## References

[1] Vanstreels RET, Kolesnikovas CKM, Sandri S, Silveira P, Belo NO, et al (2014) Outbreak of Avian Malaria Associated to Multiple Species of *Plasmodium* in Magellanic Penguins Undergoing Rehabilitation in Southern Brazil. PLoS ONE 9 4: e94994 doi:10.1371/journal.pone.0094994 24736326 10.1371/journal.pone.0094994 PMC3988135

[2] Valkiunas G (2005) Avian malaria parasites and other Haemosporidia. Boca Ratón: CRC Press. 932 p.

[3] Hellgren O, Waldenström J, Bensch S (2004) A new PCR assay for simultaneous studies of Leucocytozoon, Plasmodium, and Haemoproteus from avian blood. J Parasitol 90: 797–802. 15357072 10.1645/GE-184R1

